# Benefits of Surgical Over Medical Treatment for Unilateral Primary Aldosteronism

**DOI:** 10.3389/fendo.2022.861581

**Published:** 2022-04-26

**Authors:** Sumaiya Ahmed, Gregory L. Hundemer

**Affiliations:** ^1^ Department of Medicine, Ottawa Hospital, University of Ottawa, Ottawa, ON, Canada; ^2^ Department of Medicine (Division of Nephrology) and the Ottawa Hospital Research Institute, University of Ottawa, Ottawa, ON, Canada

**Keywords:** primary aldosteronism, hyperaldosteronism, Conn syndrome, surgery, adrenalectomy, unilateral

## Abstract

Primary aldosteronism is the most common and modifiable form of secondary hypertension. Left untreated, primary aldosteronism leads high rates of cardiovascular, metabolic, and kidney disease. Therefore, early diagnosis and targeted therapy are crucial to improve long-term patient outcomes. In the case of unilateral primary aldosteronism, surgical adrenalectomy is the guideline-recommended treatment of choice as compared to alternative medical therapies such as mineralocorticoid receptor antagonist medications. Surgical adrenalectomy is not only highly successful in reversing the biochemical abnormalities inherent to primary aldosteronism, but also in mitigating the long-term risks associated with this disease. Indeed, as opposed to medical treatment alone, surgical adrenalectomy offers the potential for disease cure. Within this review article, we review the existing evidence highlighting the benefits of surgical over medical treatment for unilateral primary aldosteronism.

## Introduction

Primary aldosteronism (PA) is a condition defined by aldosterone secretion from one or both adrenal glands, independent of renin and angiotensin II. Though historically considered a rare condition, PA is now widely recognized as the most common and modifiable form of secondary hypertension (1–5). However this condition remains highly under-diagnosed in modern-day clinical. The significance of missing this diagnosis is highlighted by the fact that left untreated, PA leads to disproportionately high rates of cardiovascular, metabolic, and kidney disease. Therefore, early disease identification and targeted therapy are crucial to reduce these risks.

The final step in the diagnostic algorithm of PA is lateralization; i.e., determining whether the source of autonomous aldosterone secretion is from the left adrenal gland, right adrenal gland, or both (6). Lateralization guides which targeted therapy is recommended to the patient. Traditionally, lifelong mineralocorticoid receptor (MR) antagonist therapy is the treatment of choice for bilateral PA. In contrast, surgical adrenalectomy (where the source of aldosterone excess is removed) is the treatment of choice for unilateral PA. Historically, the question has been raised about whether adrenalectomy is indeed superior to lifelong MR antagonist therapy in the treatment of unilateral PA. While no randomized controlled trials have served to provide this answer, a number of recent observational studies have suggested a substantial biochemical and clinical benefit of adrenalectomy over MR antagonists in the treatment of unilateral PA. Herein, we review the available evidence to date of the benefits of surgical over medical treatment for unilateral PA.

## Biochemical and Clinical Impact of Primary Aldosteronism

### Biochemical Impact of Primary Aldosteronism

PA is defined by a number of biochemical abnormalities that can be readily explained by the underlying disease pathophysiology including suppressed renin, elevated aldosterone, hypokalemia, metabolic alkalosis, and elevated glomerular filtration rate (GFR) (7). In normal physiology, aldosterone secretion is dependent upon three primary regulators: angiotensin II, potassium, and adrenocorticotropic hormone (ACTH). In contrast, PA is defined by aldosterone being secreted at high levels independent of these regulators. Aldosterone binds to and activates the MR of the principal cell of the distal nephron. MR activation leads to sodium reabsorption *via* the epithelial sodium channel (ENaC) and corresponding excretion of potassium and hydrogen ions; hence why patients often develop hypokalemia and/or metabolic alkalosis. ENaC-mediated sodium reabsorption leads to volume expansion and glomerular hyperfiltration (↑ GFR) (8) thereby leading to suppression of both renin and angiotensin II; hence why the aldosterone-to-renin ratio (ARR) is employed as a screening test as it represents high aldosterone relative to suppressed renin. Suppression of angiotensin II leads to decreased sodium reabsorption in the proximal nephron. In turn, this results in increased sodium delivery to the distal nephron which further amplifies the aldosterone-mediated sodium reabsorption *via* the ENaC channel leading to chronic volume expansion which perpetuates this vicious cycle.

### Clinical Impact of Primary Aldosteronism

The sodium retention and chronic volume expansion underlying the pathophysiology of PA along with extra-renal aldosterone-mediated MR activation lead to disproportionately high rates of cardiovascular, metabolic, and kidney disease. The extra-renal MR-mediated effects of excess aldosterone include activation of vascular endothelial and smooth muscles cells resulting in vascular fibrosis and stiffness (9–13). These extra-renal aldosterone effects help to explain why many of the long-term adverse clinical outcomes associated with PA occur even independent of blood pressure as discussed below.

It is now well-known that PA is associated with disproportionately high rates of adverse cardiovascular outcomes including coronary artery disease, congestive heart failure, atrial fibrillation, and stroke (2, 14–26). A meta-analysis which consolidated many observational studies on this topic found that PA was associated with 77% higher odds of coronary artery disease, 2-fold higher odds of congestive heart failure, 3.5-fold higher odds of atrial fibrillation, and 2.5-fold higher odds of stroke compared with essential hypertension (27). Notably, this finding of increased cardiovascular risk with PA persisted even when the analysis was restricted to only studies where patients were matched based upon blood pressure thereby highlighting the blood pressure-independent effects of PA. Several studies have further reported an increased cardiovascular mortality with PA (20, 22).

The adverse long-term health outcomes associated with PA are not limited to only cardiovascular disease. A number of adverse metabolic and kidney outcomes have also been reported. PA is associated with a higher risk of both diabetes mellitus (2, 22, 27–36) as well as metabolic syndrome (27, 29, 37) compared with essential hypertension. A meta-analysis of observational studies showed that PA was associated with 33% higher odds of diabetes mellitus and 53% higher odds of metabolic syndrome as compared with essential hypertension (27). These associations are, at least in part, related to the fact that excess glucocorticoid co-secretion often occurs along with excess aldosterone secretion in patients with PA (30). Further, the autonomous aldosterone secretion inherent to PA decreases insulin secretion and increases insulin clearance (35). In regard to kidney disease, PA has been shown to lead to early hyperfiltration followed by a steeper decline in estimated glomerular filtration rate (eGFR) along with a higher incidence of proteinuria and chronic kidney disease (CKD) (8, 38–45).

### Treatment Approach to Primary Aldosteronism

Based on the high risks of cardiovascular, metabolic, and kidney disease associated with PA, early diagnosis and targeted therapy are important to improve long-term patient outcomes. The longstanding convention for treating PA has been dependent on whether the disease is found to be unilateral or bilateral based on lateralization testing. For bilateral PA, the mainstays of treatment are dietary sodium restriction plus lifelong MR antagonist therapy (6). For unilateral PA, adrenalectomy is the recommended treatment of choice for patients healthy enough and willing to undergo surgery (6). Adrenalectomy is now typically performed *via* a laparoscopic, rather than an open, approach which has resulted in lower perioperative complication rates and shorter hospital stays (46–48). For patients with unilateral PA who are unable or unwilling to undergo adrenalectomy, the recommended treatment is the same as for bilateral PA: dietary sodium restriction plus lifelong MR antagonist therapy (6).

However, a natural question that arises from this conventional PA treatment algorithm is: ‘Does surgical adrenalectomy provide clinical benefit beyond MR antagonist therapy in the treatment of unilateral PA?’ Intuitively, adrenalectomy would be preferable in unilateral PA as it entails completely removing the source of autonomous aldosterone excess whereas MR antagonists would simply be blocking the interaction between aldosterone and the MR which is reliant on a number of factors including drug pharmacokinetics, optimal dosing, and patient compliance. However, there have been no randomized controlled trials to compare surgical versus medical therapy in unilateral PA. Such a trial has not been pursued due to the perceived lack of clinical equipoise as the longstanding notion amongst PA experts is that surgery is superior. Moreover, such a study would require the more uniform use of AVS (which is typically reserved for patients suspected of possible unilateral PA who would be amenable to surgery) to definitively evaluate disease lateralization. This would incur both increased costs to the healthcare system as well as procedure-related risks to the patient. Instead, the majority of evidence informing the benefits of surgical versus medical therapy relies upon comparing outcomes with surgical adrenalectomy for unilateral PA versus MR antagonist therapy in bilateral PA (or with unconfirmed lateralization). This comparison is somewhat confounded as unilateral and bilateral PA differ in both their underlying pathophysiology as well as in their clinical presentation. For instance, somatic mutations known to contribute to autonomous aldosterone secretion are present in the vast majority of cases of unilateral PA (49, 50). In contrast, aldosterone-producing micronodules (previously termed aldosterone-producing cell clusters), which increase in prevalence with age, lead to the autonomous aldosterone secretion underlying many cases of bilateral PA (51, 52). Additionally, unilateral PA typically presents at a younger age and with a more overt clinical phenotype than bilateral PA (2, 5, 53). Bearing these caveats in mind, the following sections summarize the existing observational evidence of the benefits of surgical adrenalectomy in the treatment of unilateral PA.

## Biochemical Outcomes Following Surgical Adrenalectomy for Unilateral Primary Aldosteronism

The biochemical changes that occur following surgical adrenalectomy for unilateral PA correlate with the physiologic sequelae that would be anticipated to arise from complete removal of the source of autonomous aldosterone excess (**Table 1**). Aldosterone levels are immediately reduced post-adrenalectomy. In turn, MR-mediated sodium reabsorption *via* the ENaC channel is reduced. The reduction in sodium reabsorption leads to volume contraction (i.e., reversal of the chronic volume expansion inherent to PA) and cessation of glomerular hyperfiltration thereby leading to ‘un-suppression’ of renin and angiotensin II. Also, with reduced MR-mediated sodium reabsorption *via* the ENaC channel, the concurrent excretion of potassium and hydrogen ions is similarly reduced. In sum, adrenalectomy for unilateral PA often results in normalization of the ARR, resolution of hypokalemia and metabolic alkalosis, and a decline in eGFR consistent with resolution of glomerular hyperfiltration. Standardized criteria defining biochemical success post-adrenalectomy have been set forth by the Primary Aldosteronism Surgery Outcomes (PASO) study (54). Below, we discuss the existing evidence for the rates of success in these biochemical outcomes post-adrenalectomy including PASO and other recent studies.

**Table 1:** Biochemical outcomes following surgical adrenalectomy for unilateral primary aldosteronism.

BIOCHEMICAL OUTCOMES FOLLOWING ADRENALECTOMY	
↓ Aldosterone	
↑ Renin	
Normalization of the Aldosterone-to-Renin Ratio (ARR)	
Resolution of Hypokalemia	
Resolution of Metabolic Alkalosis	
Reversal of Glomerular Hyperfiltration (↓ eGFR)	

### Normalization of the Aldosterone-to-Renin Ratio

The PASO study defined ‘complete biochemical success’ post-adrenalectomy as normalization of the ARR as well as correction of hypokalemia (if present pre-surgery), assessed 6-12 months post-surgery (54). Among this multi-national cohort of 699 PA patients, a striking 94% of patients met the criteria of ‘complete biochemical success’. Therefore, the vast majority of PA patients achieve a normal ARR with adrenalectomy thereby demonstrating resolution of renin-independent aldosterone secretion. The finding of a high success rate of ARR normalization with adrenalectomy has been confirmed by a number of other observational studies (55–63).

### Resolution of Hypokalemia

While hypokalemia is certainly not a universal biochemical finding in PA (64), the vast majority of unilateral PA patients who do have hypokalemia become normokalemic post-adrenalectomy. For instance, a recent Japanese retrospective cohort study of 166 unilateral PA patients demonstrated that 82% of patients who were hypokalemic prior to adrenalectomy had resolution of the hypokalemia post-operatively (59). A similar study out of the United Kingdom reported that the median serum potassium levels increased from 3.2 (IQR 2.3-4.7) mmol/L to 4.4 (IQR 3.5-5.3) mmol/L following adrenalectomy (65).

### Reduction in Glomerular Filtration Rate

As discussed above, adrenalectomy reverses the chronic glomerular hyperfiltration inherent to PA. This is reflected in decline in eGFR which should be anticipated post-adrenalectomy and usually does not indicate acute kidney injury (AKI) (8, 39–42, 44, 66). In fact, this sometimes results in CKD being ‘unmasked’ post-operatively. In a study of 25 unilateral PA patients who underwent adrenalectomy where GFR was measured pre- and six months post-adrenalectomy, the mean decline in GFR was 15 mL/min/1.73m^2^ (8). Though on the surface, this initial GFR decline post-operatively may be viewed as a negative clinical effect, it should be noted that these changes are simply hemodynamic and that longer-term kidney outcomes improve following adrenalectomy. For instance, the GFR decline in the aforementioned study (8) correlated with a concurrent decline in albuminuria, a finding that has been confirmed by a number of other observational studies (8, 39, 41, 42). Further, longitudinal eGFR decline is slowed compared to treatment with MR antagonist therapy among patients with PA. A longitudinal cohort study of 120 PA patients treated with adrenalectomy, 400 PA patients treated with MR antagonists, and a control group of 15,474 patients with essential hypertension compared rates of eGFR decline between these three groups (39). The results demonstrated that the mean annual rate of eGFR decline while similar between PA patients treated with adrenalectomy (-0.8 mL/min/1.73m^2^) and patients with essential hypertension (-0.9 mL/min/1.73m^2^) were slower than that of PA patients treated with MR antagonists (-1.6 mL/min/1.73m^2^) (39). Taking this another step further, Kobayashi et al. demonstrated that the steeper the acute fall in eGFR with PA targeted treatments, the less steep the subsequent long-term decline in eGFR (66).

## Clinical Outcomes Following Surgical Adrenalectomy for Unilateral Primary Aldosteronism

While no randomized trials on this topic exist, the clinical benefits of surgical adrenalectomy versus MR antagonist therapy (**Table 2**) have been implied by a number of observational studies. These studies must be interpreted within the limitations of their observational design including selection and referral biases, a lack of uniformity in regard to how PA is defined, and inherent pathophysiologic differences between unilateral and bilateral PA. Moreover, the fact that patients treated with MR antagonists likely reflect a mix of unilateral and bilateral PA whereas adrenalectomy is generally reserved for true unilateral PA may further add some degree of bias to these findings.

**Table 2:** Clinical outcomes following surgical adrenalectomy for unilateral primary aldosteronism.

CLINICAL OUTCOMES FOLLOWING ADRENALECTOMY	
Cure of Hypertension or Significant Improvement in Blood Pressure Control	
↓ Cardiovascular Disease Risk	
↓ Kidney Disease Risk	
↓ Diabetes Risk	
↓ Mortality	
↑ Quality of Life	

### Blood Pressure Control

The most commonly reported clinical benefits of surgical adrenalectomy for unilateral PA are in regard to blood pressure control. The PASO study defined ‘complete clinical success’ as normal blood pressure without the use of any antihypertensive medication at 6-12 months post-adrenalectomy (54). ‘Partial clinical success’ was defined as either a reduction in the number of antihypertensive medications or a reduction in blood pressure with the same number of antihypertensive medications at 6-12 months post-adrenalectomy. Among the 705 patients in the PASO study, 37% and 47% experienced complete or partial clinical success, respectively (54). Therefore, 84% of patients experienced either cure or significant improvement in blood pressure control with adrenalectomy. Reported rates of complete cure of hypertension following adrenalectomy for unilateral PA have ranged from 20-66% (55, 57, 58, 65, 67–71). These studies show that the vast majority of patients who do not achieve complete cure still have a significant improvement in blood pressure control as demonstrated by reduced blood pressure readings and/or reduced antihypertensive medication requirements. The finding that hypertension persists (though the severity is typically reduced) in many patients likely reflects the long delay, and concurrent exposure to excess aldosterone, that often exists between the timing of the onset of hypertension and the ultimate diagnosis of PA (62).

### Cardiovascular Outcomes

Observational data also suggests superiority of adrenalectomy to MR antagonist therapy in regard to cardiovascular outcomes. This was demonstrated in a retrospective study comparing 205 patients with unilateral PA treated with adrenalectomy, 602 patients with PA treated with MR antagonists, and a comparator group of >40,000 patients with essential hypertension (22). The study defined incident cardiovascular events as a composite of myocardial infarction, coronary revascularization, hospital admission for congestive heart failure, or stroke. Despite similar blood pressure control between the groups, PA patients treated with MR antagonists experienced substantially higher rates of cardiovascular events as compared with PA patients treated with adrenalectomy (HR 3.27 [95% CI 1.93-5.55]) (22). Notably, the PA patients treated with adrenalectomy experienced lower rates of cardiovascular events than even patients with essential hypertension (HR 0.58 [95% CI 0.35-0.97]) (22). Similar benefits to adrenalectomy over MR antagonist therapy for PA has also been demonstrated in atrial fibrillation (21, 26) and left ventricular hypertrophy (72, 73).

### End-Stage Kidney Disease Outcomes

In addition to the effects of adrenalectomy on eGFR discussed above, there is some evidence that adrenalectomy may lower the long-term risk of end-stage kidney disease (ESKD) compared with MR antagonist therapy. A Taiwanese study using population-wide administrative health data compared rates of ESKD between 2699 PA patients (including 657 who underwent adrenalectomy) and propensity score-matched essential hypertension patients (74). While accounting for the competing risk of death, the study found that PA patients treated with adrenalectomy had a lower risk of ESKD compared with patients with essential hypertension (sub-distribution HR 0.55, P = 0.02) (74). In contrast, PA patients treated with MR antagonists had a similar risk of ESKD compared with patients with essential hypertension (sub-distribution HR 1.08, P = 0.58) (74).

### Metabolic Outcomes

Surgical adrenalectomy is also associated with a reduced risk of incident diabetes mellitus compared with MR antagonist therapy. A longitudinal population-based Taiwanese study of 2,367 PA patients without diabetes mellitus at baseline found that targeted therapy with an MR antagonist was associated with an increased risk of incident diabetes mellitus (HR 1.16, P < 0.001) whereas targeted therapy with adrenalectomy was associated with a reduced risk of incident diabetes mellitus (HR 0.61, P < 0.001) (31). These associations were confirmed in a large United States observational study (22). The reduction in diabetes risk with adrenalectomy may be related to the fact that cortisol is frequently co-secreted from aldosterone producing adenomas (30, 75). Indeed, targeted treatment in PA is associated with increased insulin sensitivity and decreased insulin clearance (35).

### Mortality

The aforementioned Taiwanese population-based studies also suggest a reduction in mortality among patients with unilateral PA treated with adrenalectomy compared with patients with essential hypertension (23, 74). Chen et al. reported no significant difference in mortality comparing PA treated with MR antagonists versus essential hypertension (HR 1.05 [95% CI 0.94-1.14]) (74). In contrast, PA treated with adrenalectomy was associated with a reduced risk in mortality compared with essential hypertension (HR 0.22 [95% CI 0.15-0.34]) (74).

### Quality of Life

While both adrenalectomy and MR antagonists improve quality of life measures for patients with PA, adrenalectomy has been shown to improve these measures both more rapidly and more robustly (63, 76–78). For instance, two Australian studies examined changes in quality of life among patients with unilateral PA treated and patients with PA treated with spironolactone or amiloride (63, 76). At the time of diagnosis and prior to targeted treatment, patients with PA had lower quality of life scores as assessed by the Medical Outcomes Study Short Form 36 General Health Survey (SF-36). While quality of life measures were seen to improve within three months among patients with unilateral PA treated with adrenalectomy (63), improvement was not seen until six months among patients with bilateral PA treated with medical therapy (76). Notably, the degree of improvement in quality of life seen after six months for bilateral PA treated with medical therapy was lower than that seen within the same time frame for unilateral PA treated with adrenalectomy (63, 76).

## Conclusion

**Figure 1:**
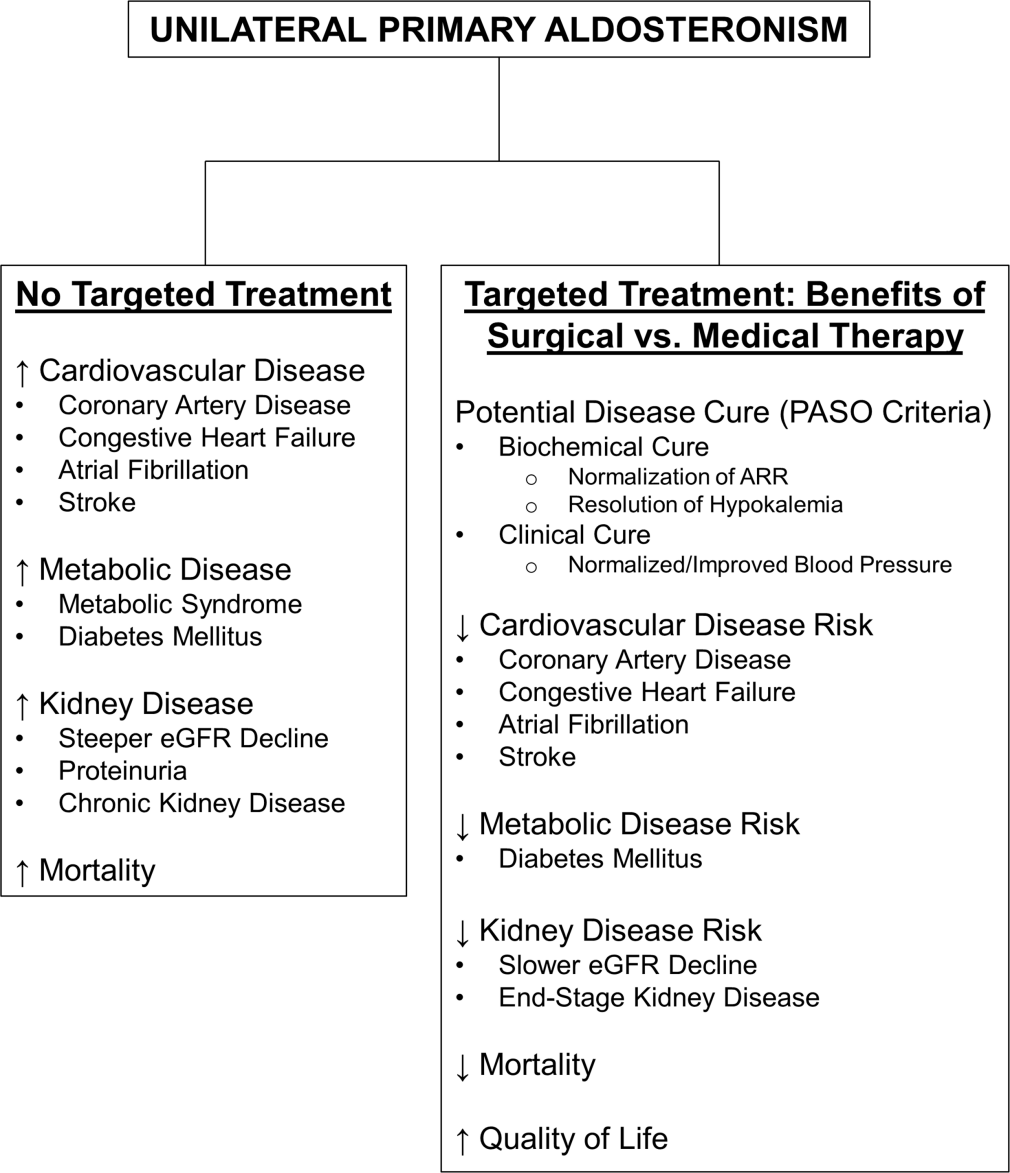
Summary of the Health Impact of Untreated Primary Aldosteronism and the Benefits of Surgical vs. Medical Therapy in the Treatment of Unilateral Primary Aldosteronism. ARR, aldosterone-to-renin ratio; eGFR, estimated glomerular filtration rate; PASO, Primary Aldosteronism Surgery Outcomes study (54).

Early diagnosis and targeted treatment for PA are critical given the disproportionate morbidity and mortality linked with this condition. Moreover, identifying disease lateralization early in the diagnostic algorithm is necessary to provide optimal, personalized care for PA patients. Guidelines recommend surgical adrenalectomy over medical therapy with MR antagonists for patients with unilateral PA who are healthy enough and willing to undergo surgery. In addition to a high degree of success in reversing the biochemical abnormalities associated with PA, a key benefit of adrenalectomy over MR antagonists for unilateral PA is the potential for disease cure as the source of autonomous aldosterone excess is fully removed rather than its effect simply being blocked. More importantly, despite biases inherent to the existing observational literature, the sheer abundance of evidence suggesting improved clinical outcomes with adrenalectomy versus MR antagonists continues to mount. These include significant reductions in blood pressure, cardiovascular events, kidney disease, diabetes mellitus, and mortality along with improvements in quality of life as summarized in **Figure 1**. As a whole, these findings strongly support the current guideline recommendations advising surgical adrenalectomy to treat unilateral PA.

## Author Contributions

Conception and design of the review and drafting of the manuscript: SA and GH. All authors contributed to the article and approve the submitted version.

## Funding

GH is supported by the Canadian Institutes of Health Research Institute of Nutrition, Metabolism and Diabetes (Reference # PJT-175027), the Kidney Foundation of Canada (Reference # 851937-21KHRG), the Kidney Research Scientist Core Education and National Training (KRESCENT) Program New Investigator Award (Reference # 2019KP-NIA626990), and the Lorna Jocelyn Wood Chair for Kidney Research.

## Conflict of Interest

The authors declare that the research was conducted in the absence of any commercial or financial relationships that could be construed as a potential conflict of interest.

## Publisher’s Note

All claims expressed in this article are solely those of the authors and do not necessarily represent those of their affiliated organizations, or those of the publisher, the editors and the reviewers. Any product that may be evaluated in this article, or claim that may be made by its manufacturer, is not guaranteed or endorsed by the publisher.
